# Gluten restriction in irritable bowel syndrome, yes or no?: a GRADE-assessed systematic review and meta-analysis

**DOI:** 10.3389/fnut.2023.1273629

**Published:** 2023-11-01

**Authors:** Erfan Arabpour, Dorsa Alijanzadeh, Amir Sadeghi, Sina Khoshdel, Azita Hekmatdoost, Hamed Kord-Varkaneh, Mohammad Abdehagh

**Affiliations:** ^1^Gastroenterology and Liver Diseases Research Center, Research Institute for Gastroenterology and Liver Diseases, Shahid Beheshti University of Medical Sciences, Tehran, Iran; ^2^Department of Clinical Nutrition and Dietetics, Faculty of Nutrition and Food Technology, National Nutrition and Food Technology, Research Institute, Shahid Beheshti University of Medical Sciences, Tehran, Iran; ^3^Department of Nutrition, School of Medicine, Hamadan University of Medical Sciences, Hamadan, Iran

**Keywords:** gluten, irritable bowel syndrome, GFD, IBS, meta-analysis

## Abstract

**Background:**

More than half of patients with irritable bowel syndrome (IBS) report aggravating their symptoms with certain foods. Currently, Low fermentable oligo-, di-, and monosaccharides and polyols diet (LFD) is the most accepted dietary intervention for IBS. Recent randomized controlled trials (RCTs) have been suggested that gluten restriction may reduce the symptoms of patients with IBS. However, the results from these studies are conflicting. This study filled this knowledge gap by evaluating the impact of the gluten-free diet (GFD) on IBS symptoms.

**Methods:**

A systematic search was carried out in Pubmed/Medline, Cochrane CENTRAL, Scopus, and Web of Science up to April 2023. A random-effect model was applied to estimate the standardized mean difference (SMD) and 95% confidence interval (95% CI) for each outcome.

**Results:**

A total of nine controlled trials were included in the meta-analysis. In contrast to gluten-containing diet, GFD was unable to reduce overall symptoms (SMD − 0.31; 95% CI −0.92, 0.31), bloating (SMD −0.37; 95% CI −1.03, 0.30), and quality of life (SMD −0.12, 95% CI −0.64, 0.39); but had a slight trend to reduce abdominal pain (SMD –0.68; 95% CI −1.36, −0.00). Also, LFD significantly reduced the IBS-Severity score system (SMD 0.66, 95% CI 0.31, 1.01) and improved quality of life (SMD −0.36, 95% CI −0.70, −0.01), compared to GFD.

**Conclusion:**

A GFD is not robust enough to be routinely recommended for IBS patients, and its efficacy is significantly lower than that of an LFD. Only a certain subgroup of IBS patients may benefit from GFD; further studies are needed to target this subgroup.

## 1. Introduction

Irritable bowel syndrome (IBS) is a chronic symptom-based disorder of the gastrointestinal (GI) tract. Patients experience symptoms of altered bowel habits, with either constipation, diarrhea, or both, and abdominal pain. Based on pool prevalence analysis, it is estimated that the worldwide prevalence of IBS is 11.2%; however, this might vary from 1.1 to 45.0% according to the criteria used and the country (1). Currently, different first-line therapies, including exercise and pharmacologic options for managing symptoms, are recommended (2).

Even though patients benefit from pharmacological therapies, the results of surveys are indicative of patients’ tendency to dietary modifications, more specifically, foods containing high carbohydrates and fat (3), as 63% reported that their symptoms could be triggered by eating certain foods (4). Some studies suggest that patients with IBS may be intolerant to gluten in the absence of celiac disease (5), which is termed non-celiac gluten sensitivity (NCGS). In fact, patients report benefiting from avoiding gluten from their diet. It is also said that gluten reintroduction might worsen symptoms (6). The possibility that a subset of patients with IBS could fall into NCGS indicates the need to investigate the actual effect of a gluten-free diet (GFD) on the patients (7). Previously, it was shown that the evidence to recommend a GFD to reduce symptoms of patients with IBS is insufficient; although a GFD was associated with a reduced risk of experiencing overall symptoms, but this reduction was not statistically significant (8). So, the recent IBS guidelines do not recommend GFD in IBS. However, due to insufficient data, more trials comparing GFD head-to-head with a Low fermentable oligo-, di- and monosaccharides and polyols diet (LFD) are recommended (9).

The previous systematic review and meta-analysis in this field had some limitations and included merely two randomized controlled trials (RCTs) evaluating the effect of a GFD. Moreover, the analysis result of the included studies with GFD was only compared to a gluten-containing diet (GCD) (8). Since the publication of this study, there have been additional RCTs evaluating different aspects of gluten restriction in IBS. This study aims to update the impact of a GFD on GI symptoms and the quality of life of patients with IBS and compare its efficacy with an LFD to provide clinicians and dieticians an evidence-based assessment of diet therapy in IBS.

## 2. Methods

This study was conducted and reported according to the 2021 updated Preferred Reported Items for Systematic Reviews and Meta-analysis (PRISMA) statement (10).

### 2.1. Search strategy

We searched Pubmed/Medline, Cochrane CENTRAL, Scopus, and Web of Science for studies reporting the efficacy/effectiveness of GFD in patients with IBS, published up to April 27, 2023. The search terms were gluten, gluten-free, GFD, irritable bowel syndrome, IBS, irritable colon, and spastic colon (Supplementary Table S1). No language restrictions were imposed.

### 2.2. Study selection and eligibility criteria

The records found through database searching were merged, and the duplicates were removed using EndNote X20. Two authors independently screened the records by title/abstract and full texts to exclude those unrelated to the study.

All the eligible studies that were included in our analysis according to the PICOS strategy as follows: (1) Population: patients older than 16 years and with the diagnosis of IBS based on ROME III/IV criteria and exclusion of the celiac disease; (2) Intervention: elimination or restriction of gluten in daily diet; (3) Comparators: comparison with placebo, low-FODMAP diet, regular diet, or any non-gluten restricted diet(s); (4) Outcome: those which reported mean changes and their standard deviations (SDs) of GI symptoms including overall symptoms, abdominal pain, bloating, nausea, tiredness, satisfaction with stool consistency, and also quality of life, over the length of the study for both intervention and control groups; and (5) Study design: having a parallel or cross-over design in an RCT setting. Conference abstracts, reviews, experimental studies on animal models, and articles that their full-text or original data were not available were excluded.

### 2.3. Data extraction

A pre-specified Excel form was used to extract data from the included studies. Two independent reviewers extracted the following items from all eligible studies: first author’s name, publication year, country/ies where the research was conducted, study design, patients’ demographics (age and sex), IBS diagnostic criteria, celiac exclusion methods, prior diet before study initiation, treatment protocols, methodology, sample size, and mean changes and their SDs of all the mentioned outcomes. Data were inserted into Excel sheets, and any differences or disagreements were resolved by consensus or a third reviewer.

### 2.4. Assessment of risk of bias and GRADE methodology

The methodological quality of included trials was assessed using the Cochrane risk of bias tool on a domain-based evaluation in this meta-analysis (11). The overall evidence across the studies was sorted following the GRADE (Grading of Recommendations Assessment, Development, and Evaluation) methodology using the GRADEPro guideline development tool (GDT) (12).

### 2.5. Data synthesis and statistical analysis

In the GFD and control groups, mean and SD changes were applied for each variable to acquire the related effect sizes. If no SD changes were reported, they were calculated by taking into account the changes in the concentration of each variable during the trial. 95% confidence intervals (CIs), Interquartile ranges (IQRs), and standard errors (SEs) were converted to SDs (13). We also used a random-effects model that took into account variations between studies to get the overall effect sizes. *I*^2^ statistic test was applied for heterogeneity determination. *I*^2^ value>50% was characterized as significant heterogeneity between studies (14). Subgroup analyses were performed to find probable sources of heterogeneity based on the pre-defined variables such as intervention length (4 ≥ vs. >4 weeks), gluten dose (elimination vs. restriction), risk of bias (low risk vs. high risk of bias), and subtype of IBS (constipation, diarrhea, or mixed). Influence analysis was performed to determine if the overall effect size depended upon a certain study (15). Publication bias was evaluated statistically using Egger’s regression test and the funnel plot if more than five studies were identified (16). The meta-analysis was conducted using the STATA® version 17.0 (StataCorp, College Station, Lakeway, TX, United States). *p* value <0.05 was considered a significant level.

## 3. Results

**Figure 1:**
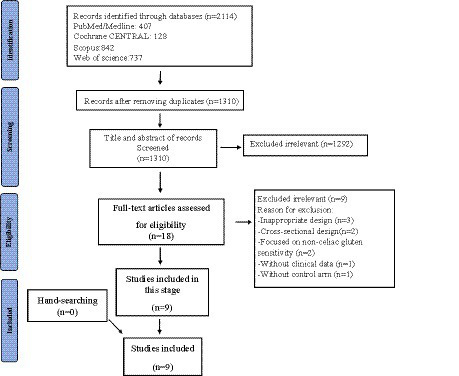
Flowchart of study selection for inclusion trials in the systematic review.

We investigated a total of 1,310 records found in the systematic search; after removing duplicates and full-text reviews, nine were chosen. Studies included and excluded through the review process are summarized in Figure 1; Supplementary Table S2. Among the included studies, there were six parallel and two cross-over RCTs and one non-randomized controlled trial. The studies originated from six countries: Iran (*n* = 4), Sweden (*n* = 2), and Italy, United Kingdom, and India (*n* = 1, for each one). The complete information on the included studies is shown in Table 1.

**Table 1:** Characteristics of included studies.

Reference	Country	Study design	Participants	Prior diet before study initiation	Intervention	Gluten dose	Methodology	Duration of therapy	IBS definition	Predominant stool type	Outcome	Celiac exclusion method
Shahbazkhani (17)	Iran	Double-blind RCT	Adults age > 16 years with newly diagnosed IBS	GFD^a^	Patients received packages containing gluten or gluten-free powder	Not measured	Block randomization method, patients and investigators were both blinded	12 weeks	ROME III	IBS-D, IBS-C, and IBS-M	Primary outcome was systematic improvement and scored with VAS	Serology and histology
Algera (18)	Sweden	Double-blind cross-over RCT	Adult sex- and aged-matched IBS patients	Regular diet	Patients received gluten (14 g/day) or rice flour powder over their meals	14 g/day	Randomization by drawing concealed envelops by a nurse not involved in the study, did not mention allocation ratio, double blinded	2 weeks	ROME IV	IBS-D, IBS-C, and IBS-M	Primary outcomes changes in IBS-SSS and bowel habits. Secondary outcomes changes in fecal microbiota and metabolite profile	Serology
Hajiani (19)	Iran	Double-blind RCT	Rome III diagnosed IBS patients who did not respond to prior treatments	Regular diet	GFD versus regular diet for 12 weeks	Not measured	Manual randomization (based on odd and even days) did not mention allocation ratio, patients and investigators were both blinded	12 weeks	ROME III	Not specified	Changes in overall symptoms and bowel habits	Not mentioned
Nordin (20)	Sweden	Three-way cross-over RCT	Adults with moderate to severe IBS (IBS-SSS score > 175) based on ROME IV criteria	Regular diet	1 week interventions with FODMAPs (50 g/day), gluten (17.3 g/day), or placebo, separated by 1 week washout	17.3 g/day	Block randomization method, patients and investigators were both blinded	1 week	Rome IV	IBS-D, IBS-C, and IBS-M	Changes in IBS-SSS and GI symptoms	Not mentioned
Paduano (21)	Italy	Non-randomized non-blind clinical trial	Adults aged between 18 and 45 years with newly diagnosed IBS	Regular diet	LFD, GFD, or balanced Mediterranean diet	Not measured	No randomization, no blinding	4 weeks	Rome IV	IBS-D, IBS-C, and IBS-M	Changes in IBS-SSS, quality of life, and GI symptoms evaluated by VAS	Not mentioned
Rej (22)	United Kingdom	Non-blind RCT	Adults aged 18 years with Rome IV IBS-D, or IBS-M, and IBS-SSS > 75	Regular diet	Traditional dietary advice, LFD, or GFD for 4 weeks	Not measured	Computer-generated block-randomization in 1:1:1 allocation ratio, no blinding	4 weeks	ROME IV	Non-constipated IBS	Primary outcome response to dietary intervention (>50 reductions in IBS-SSS), secondary outcomes included changes in individual IBS-SSS items within clinical responders, acceptability and food-related quality of life with dietary therapy, changes in nutritional intake, and alterations in stool dysbiosis index	Serology
Saadati (23)	Iran	Single-blind RCT	Adults aged 18–80 years with IBS based on ROME IV criteria	GFD+ LFD	GFD + LFD vs. restricted gluten(8,16, and 32 g/day) vs. unrestricted diet (normal diet)	8, 16, and 32 g/day	Randomization method unclear, did not mention allocation ratio, patients were blinded to the treatment	6 weeks (and 2 weeks)	ROME IV	IBS-D, IBS-C, and IBS-M	Changes in quality of life and GI symptoms evaluated by VAS	Serology and histology
Zanwar (24)	India	Double-blind RCT	Patients aged >16 years, with symptoms of IBS as per the Rome III criteria	GFD^a^	Gluten group consumed two slices of bread containing gluten, placebo group consumed two slices of gluten-free bread	Not measured	Computer-generated randomization, did not mention allocation ratio, patients and investigators were both blinded	4 weeks	ROME III	Not specified	Changes in overall symptoms and individual GI symptom by VAS	Serology and histology
Mohseni (25)	Iran	Double-blind RCT	Adults aged between 18 and 65 years with IBS based on ROME IV criteria	Regular diet	Intervention group received an LFD with 5 g/day rice flour, Control group received 5 g/day gluten powder	5 g/day	Randomization method unclear, did not mention allocation ratio, patients and investigators were both blinded	6 weeks	ROME IV	IBS-D, IBS-C, and IBS-M	The primary outcome was a significant reduction of the IBS-SSS. Secondary endpoints were changes in IBS symptoms and quality of life	Not mentioned

GFD, Gluten free diet; GI, Gastrointestinal; IBS-C, D, or M, IBS-constipation, diarrhea, or mixed; IBS-SSS, IBS-severity score system; LFD, Low FODMAP; and VAS, Visual analog scale. ^a^Patients who responded to this initial diet were included in the study.

### 3.1. GFD vs. GCD

#### 3.1.1. Overall symptoms

**Figure 2:**
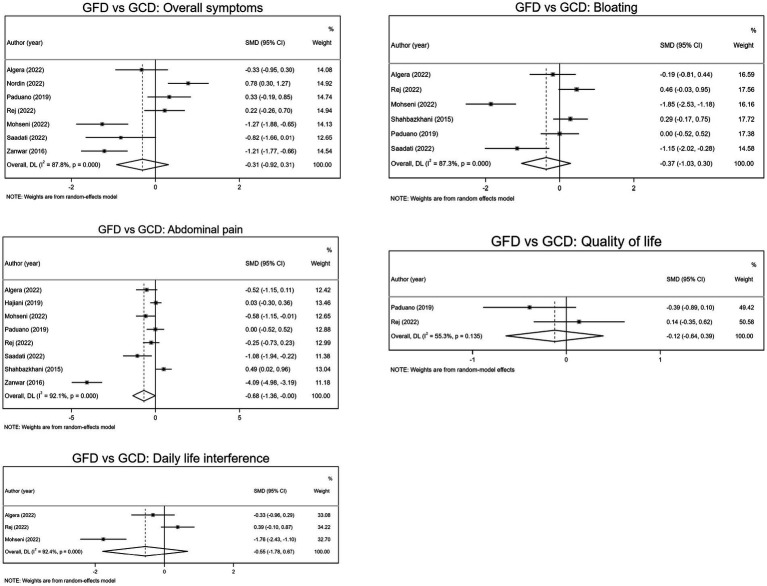
Forest plots of GFD vs. GCD.

Seven trials evaluated overall symptoms as an outcome measure (intervention samples = 186/ control samples = 182). GFD resulted in a reduction of overall symptoms (SMD − 0.31; 95% CI −0.92, 0.31). Although this was not statistically significant. Subgroup analysis showed that overall symptoms significantly subsided after a period of more than 4 weeks on GFD (SMD −1.11; 95% CI −1.61, −0.61), whereas symptoms did not subside significantly in those who followed the diet for less than 4 weeks (SMD −0.03; 95% CI −0.70, 0.64; Table 2; Figure 2).

**Table 2:** Subgroup analysis of GFD vs. GCD effect on gastrointestinal symptoms.

Outcome and subgroups	Number of studies	SMD (95% CI)	Heterogeneity		
			*p* heterogeneity	*I* ^2^	*p* between
*Abdominal pain*					
*Overall effect*	8	−0.68 (−1.36, −0.00)	<0.001	92.1%	-
*Trial duration*					
Up to 4 weeks	4	−1.17 (−2.57, 0.23)	<0.001	95.4%	0.210
More than 4 weeks	4	−0.20 (−0.77, 0.36)	0.003	78.8%	
Risk of bias					
Low risk of bias	5	−0.40 (−0.71, −0.09)	0.234	28.2%	0.450
High risk of bias	3	−1.13 (−3.01, 0.75)	<0.001	97.6%	
*Overall symptoms*					
*Overall effect*	7	−0.31 (−0.92, 0.31)	<0.001	87.8%	-
*Trial duration*					
Up to 4 weeks	5	−0.03 (−0.70, 0.64)	<0.001	87.4%	0.011
More than 4 weeks	2	−1.11 (−1.61, −0.61)	0.403	0.0%	
*Bloating*					
*Overall effect*	6	−0.37 (−1.03, 0.30)	<0.001	87.3%	-
*Trial duration*					
Up to 4 weeks	3	0.12 (−0.26, 0.50)	0.228	32.3%	0.182
More than 4 weeks	3	−0.88 (−2.31, 0.54)	<0.001	93.0%	

Influence analysis was done and did not indicate any evidence of sensitivity (Supplementary Table S3).

#### 3.1.2. Abdominal pain

Pooled effect sizes from eight trials (intervention samples = 258/control samples = 252) indicated GFD significantly reduced abdominal pain compared to GCD (SMD –0.68; 95% CI −1.36, −0.00), while subgroup analysis showed no significant improvement of abdominal pain, regardless of the length of the trial. Also, subgroup analysis showed a significant reduction of abdominal pain in low risk of bias studies (SMD −0.40, 95% CI −0.71, −0.09), while no significant effect for high risk of bias studies (SMD −1.13; 95% CI −3.01, 0.75; Table 2; Figure 2).

Influence analysis indicated that the outcomes lack statistical significance when the exclusion of the five trials is taken into account (Supplementary Table S4).

#### 3.1.3. Bloating

As indicated in Figure 2, pooled data from six studies (intervention samples = 158/control samples = 152) showed no significant reduction of bloating with GFD compared to GCD (SMD −0.37; 95% CI −1.03, 0.30). Additionally, subgroup analyzes showed that neither gluten restriction for less than 4 weeks nor for more than 4 weeks reduced bloating (Table 2).

Influence analysis was done and did reveal any evidence of sensitivity (Supplementary Table S5).

#### 3.1.4. Quality of life

Two controlled trials evaluated quality of life as an outcome measure (intervention samples = 63/control samples = 67). The pooled data suggested GFD is unable to improve the quality of life of the patients with IBS (SMD −0.12, 95% CI −0.64, 0.39; Figure 2).

#### 3.1.5. Daily life interference

Pooled data from three RCTs (intervention samples = 76/control samples = 79) showed no significant reduction of bloating with GFD compared to GCD (SMD −0.55; 95% CI −1.78, 0.67; Figure 2).

### 3.2. GFD vs. LFD

#### 3.2.1. Total IBS-SSS

**Figure 3:**
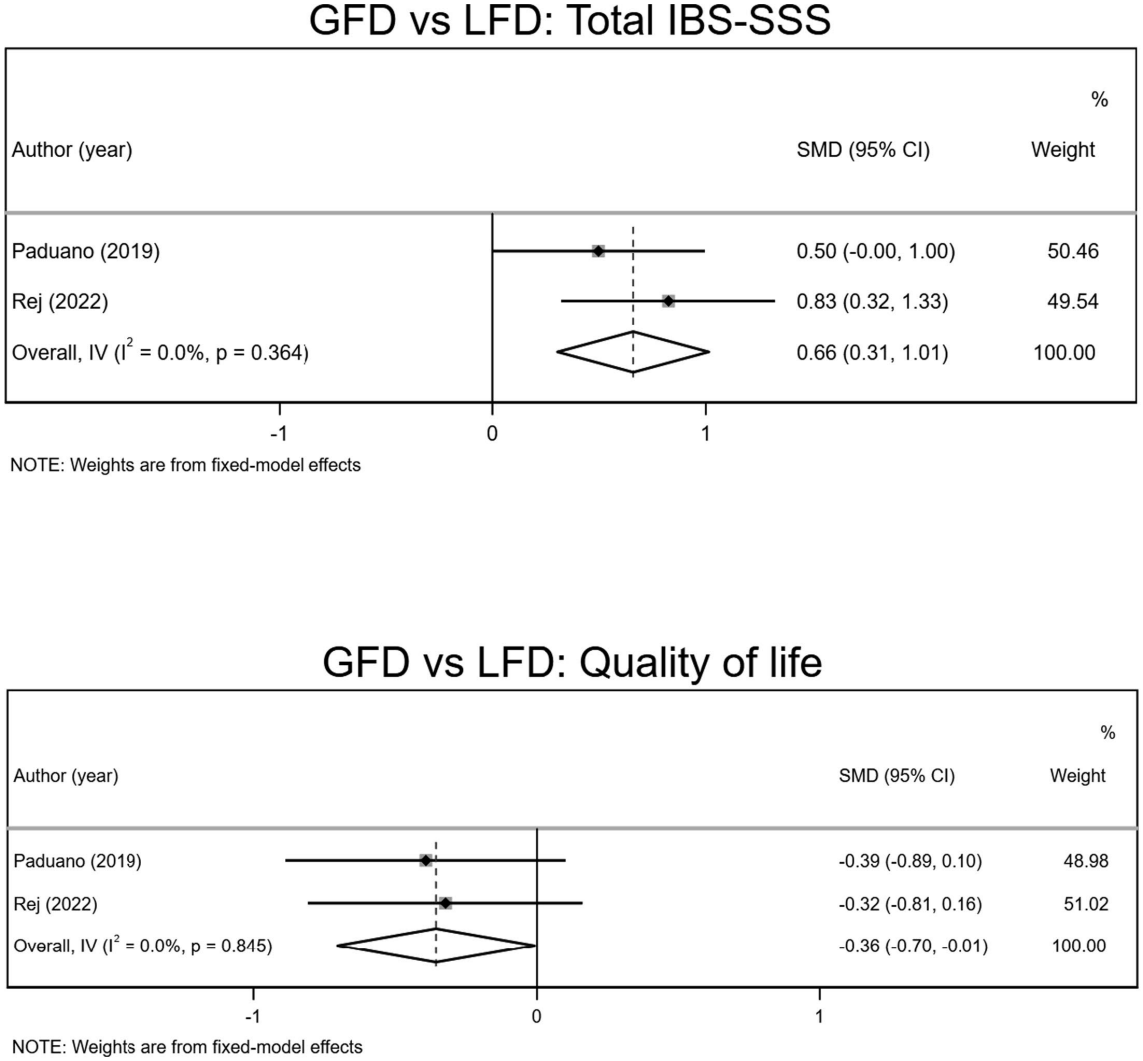
Forest plots of GFD vs. LFD.

As indicated in Figure 3, pooled data from two trials (intervention samples = 63/control samples = 67) showed LFD significantly reduced IBS-SSS, compared to GFD (SMD 0.66, 95% CI 0.31, 1.01).

#### 3.2.2. Quality of life

Two studies evaluated quality of life as an outcome measure (intervention samples = 63/control samples = 67). The pooled data suggested LFD significantly improves the quality of life of the patients with IBS, compared to GFD (SMD −0.36, 95% CI −0.70, −0.01; Figure 3).

### 3.3. Publication bias

We used Egger’s regression test and funnel plots to assess the possibility of publication bias if more than five studies were identified. There was a significant publication bias for abdominal pain (*p* = 0.035). However, no evidence of publication bias was observed for reports evaluating the influences of GFD on overall symptoms (*p* = 0.151), and bloating (*p* = 0.054). The funnel plots also proved these findings (Supplementary Figure S1).

### 3.4. Risk of bias and GRADE

According to the Cochrane quality assessment tool, six studies had a low risk of bias, while three studies had a high risk of bias (Supplementary Table S6). An assessment of the quality of evidence using the GRADE criteria is presented in Tables 3, 4. The overall quality of the evidence was “very low” for GFD vs. GCD and “low” for GFD vs. LFD, as there were serious or very serious limitations in the terms of risk of bias, inconsistency, imprecision, and publication bias.

**Table 3:** GRADE profile of GFD vs. GCD.

Outcome	Number of studies	Study design	Risk of bias	Inconsistency	Indirectness	Imprecision	Other considerations	Number of GFD/GCD	Certainty	Importance
Overall symptoms	7	Randomized	Serious^a^	Very serious^b^	Not serious	Serious^d^	All plausible residual confounding would reduce the demonstrated effect	186/182	⨁◯◯◯ Very low	Critical
Abdominal pain	8	Randomized	Serious^a^	Very serious^b^	Not serious	Serious^d^	Publication bias/ all plausible residual confounding would reduce the demonstrated effect	258/252	⨁◯◯◯ Very low	Critical
Bloating	6	Randomized	Serious^a^	Very serious^b^	Not serious	Serious^d^	All plausible residual confounding would reduce the demonstrated effect	158/152	⨁◯◯◯ Very low	Critical
Quality of life	2	Randomized	Not serious	Serious^c^	Not serious	Serious^d^	Publication bias is strongly suspected/ all plausible residual confounding would reduce the demonstrated effect	63/67	⨁◯◯◯ Very low	Critical
Daily life interference	2	Randomized	Not serious	Very serious^b^	Not serious	Serious^d^	Publication bias is strongly suspected/all plausible residual confounding would reduce the demonstrated effect	76/79	⨁◯◯◯ Very low	Critical

^a^High risk of bias due to the random generation sequencing, selective reporting, incomplete outcome data, and other sources of bias. ^b^There is high heterogeneity for overall symptoms (*I*^2^ = 87.8%), abdominal pain (*I*^2^ = 92.1%), bloating (*I^2^* = 87.3%), and daily life interference (*I^2^* = 92.4%). ^c^There is moderate heterogeneity for quality of life (*I*^2^ = 55.3%). ^d^95% CI including 0, the effect estimate comes from a few number of studies.

**Table 4:** GRADE profile of GFD versus LFD.

Outcome	Number of studies	Study design	Risk of bias	Inconsistency	Indirectness	Imprecision	Other considerations	Number of GFD/LFD	Certainty	Importance
IBS-SSS	2	Randomized	Not serious	Not serious	Not serious	Serious^a^	Publication bias is strongly suspected/all plausible residual confounding would reduce the demonstrated effect	63/67	⨁⨁◯◯ Low	Critical
Quality of life	2	Randomized	Not serious	Not serious	Not serious	Serious^a^	Publication bias is strongly suspected/all plausible residual confounding would reduce the demonstrated effect	63/67	⨁⨁◯◯ Low	Critical

^a^The effect estimate comes from a few number of studies.

## 4. Discussion

In this study, we aimed to compare the efficacy of GFD to GCD and LFD in IBS. As shown, compared to GCD, GFD was unable to reduce overall symptoms, bloating, and quality of life, but it had a slight trend to reduce abdominal pain. However, it seems even this reduction in abdominal pain is also not much reliable, as the sensitivity analysis showed the results are strongly influenced by some trials. On the other hand, compared to GFD, LFD significantly improved patients’ symptoms and quality of life. These results suggest that, unlike an LFD, a GFD cannot be a routine recommendation for IBS patients.

Gluten-related GI disorders can be categorized as wheat allergy, celiac disease, or NCGS. NCGS is characterized by the distress following gluten consumption in the absence of celiac disease and IgE-mediated allergy (26). This condition is known to be an independent clinical condition and is associated with IBS (27). Notably, most individuals seeking medical options for gluten-induced GI symptoms are found to have no association with celiac disease or wheat allergy (28). As Kaukinen et al. reported, among 94 adults with abdominal symptoms following cereal ingestion, 9% were diagnosed with celiac disease, 8% with latent celiac, and 20% with cereal allergy. Thus, 63% of patients could not be in either the celiac or allergic group; however, they were affected by gluten foods and clinically benefitted from a GFD (29). Given the relatively high prevalence of anti-gliadin antibodies among patients with IBS, a suggested hypothesis was to introduce a subgroup of IBS who experience symptoms most likely manifested by anti-gliadin antibody production. These patients will experience symptom relief with gluten restriction (28, 30).

Importantly, it is shown that gut microbiota is under the influence of GFD, and fecal metabolites of microbiota could be a predictor of participants’ response to GFD (18). In line with these findings, a study by Dieterich et al. showed that Bacteroidetes numbers had a significant increase in patients on a GFD for 2 weeks in comparison with patients on an LFD. The microbial diversity and imbalance in NCGS can be a potential etiology of the patients’ symptoms (31). In fact, the gut microbiota is in dynamic interaction with the immune system, and a balanced microbiota enhances immune responses (32). A strong body of evidence demonstrated the role of inflammation and gluten-triggered immune reactions in GI symptoms of patients with IBS (31, 33). Low-grade inflammation is reported in biopsies from colonic mucosa of more than half of the patients with IBS, which is indicative of food hypersensitivity (34). The increased mast cells as well as increased expression of toll-like receptors (TLRs) in GI mucosa of NCGS patients, are evidences of immune system involvement (35). Furthermore, anti-gliadin immunoglobulin G (IgG) antibodies, which are reported to be predictors of response to GFD (30), are found in more than half of the NCGS patients, thus further supports the suggestion of an immune-mediated mechanism being involved (36). Limited gluten consumption through directly alleviating the pro-inflammatory state, as well as modulating the gut microbiota to further interact with the immune system could result in patients benefiting from GFD (24).

There is an extensive debate about the possible mechanisms by which GFD might impact IBS symptoms. It is suggested that symptoms of IBS patients are more likely to be induced by fructan rather than gluten (25, 37), and barley and wheat exacerbate the patients’ symptoms mainly due to their fructan content, while gluten might be responsible for symptoms of a few percent of subjects (25). As an RCT by Skodje et al. (37) showed, a gluten challenge resulted in no significant difference compared to placebo and fructan in patients’ symptoms, and a small daily amount of fructan, as fructan challenge, increased GI symptoms of participants. The results can be explained by the fact that many foods containing gluten, which is restricted in GFD, also contain fructan (38). However, the result of a food diary study by Algera and colleagues was not in agreement with these findings, as they found no association between fructan intake and the severity of GI symptoms in patients with IBS (39).

Interestingly, it is reported that patients with IBS who experienced more severe symptoms consumed less gluten and calories (39), but caution must be taken in drawing conclusions. Patients might avoid foods containing gluten and, as a result, consume fewer calories. Given that patients with IBS show more tendency to cut calorie intake, dietary counseling is also important in these individuals, especially regarding nutritional inadequacies (40).

To further assess the concept of IBS and NCGS overlap, we aimed to examine the hypothesis that subjects with IBS experience GI symptoms in a dose-dependent way. However, due to the heterogeneity of studies, we could not perform a dose–response analysis regarding the amount of dietary gluten and its potential association with patients’ symptoms. Nevertheless, current evidence supports no significant dose influence of gluten on IBS subjects. The studies by Saadati et al. (23) and Biesiekierski et al. (41) have evaluated gluten restriction. The authors of both trials did not find a dose-dependent pattern of the effects of gluten challenge on the symptoms of subjects with IBS. Even though, currently, no solid evidence has been provided to explain the possible mechanism, we believe that this might be explained through the fact that IBS is a large heterogeneous group consisting of individuals with possibly different etiologies which impose them to IBS symptoms. Thus, it would be rational to consider a subgroup as gluten-sensitive that might be responsive to gluten restriction (28).

Even though GFD might show limited improvement in particular symptoms of patients, it is noteworthy that in many regions, gluten-free products might not be widely available or financially affordable, which makes this dietary change seem to be inconvenient for some patients, thus might reduce patients’ adherence to the GFD. As in some included trials, an innegligible number of patients left the study as they were unable to cope with the dietary restrictions. A non-blinded RCT compared the impact of three types of diet, including the traditional dietary advice, GFD, and LFD, on subjects with non-constipation IBS. The results showed traditional dietary advice to have similar efficacy with LFD and GFD in non-constipated subjects with IBS. Given its accessibility and cost, the traditional diet is recommended as the first choice for this category of IBS patients. Furthermore, GFD and LFD were more expensive and more time-consuming to be prepared for subjects compared to the traditional dietary advice, thus had less acceptability, and the proportion of individuals who considered continuing a GFD was reported to be less than individuals who accepted LFD and these two diets were both less acceptable than traditional dietary advice (22). However, subjects’ adherence to GFD in another study was found to be high as the one-year follow-up showed all responders and 55% of non-responders were continuing GFD. Therefore, it can be assumed that patients’ diet adherence depends on the accessibility to GFD, which makes different studies report different rates (42).

Another controversy about GFD is the time required to see its effectiveness on IBS symptoms. In suspected cases of NCGS, the Salerno criteria recommends 6 weeks of GFD (43), but there is no agreement on the IBS settings. In our included studies, the length of the trials was 1–12 weeks. Considering the number of the studies and their length of intervention, studies were divided into two subgroups of less than 4 weeks and more than 4 weeks. Although the pooled data from eight studies showed a trend of reduction of abdominal pain, but this significance was lost for both subgroups, without between subgroup heterogeneity (*p* = 0.210).

**Figure 4:**
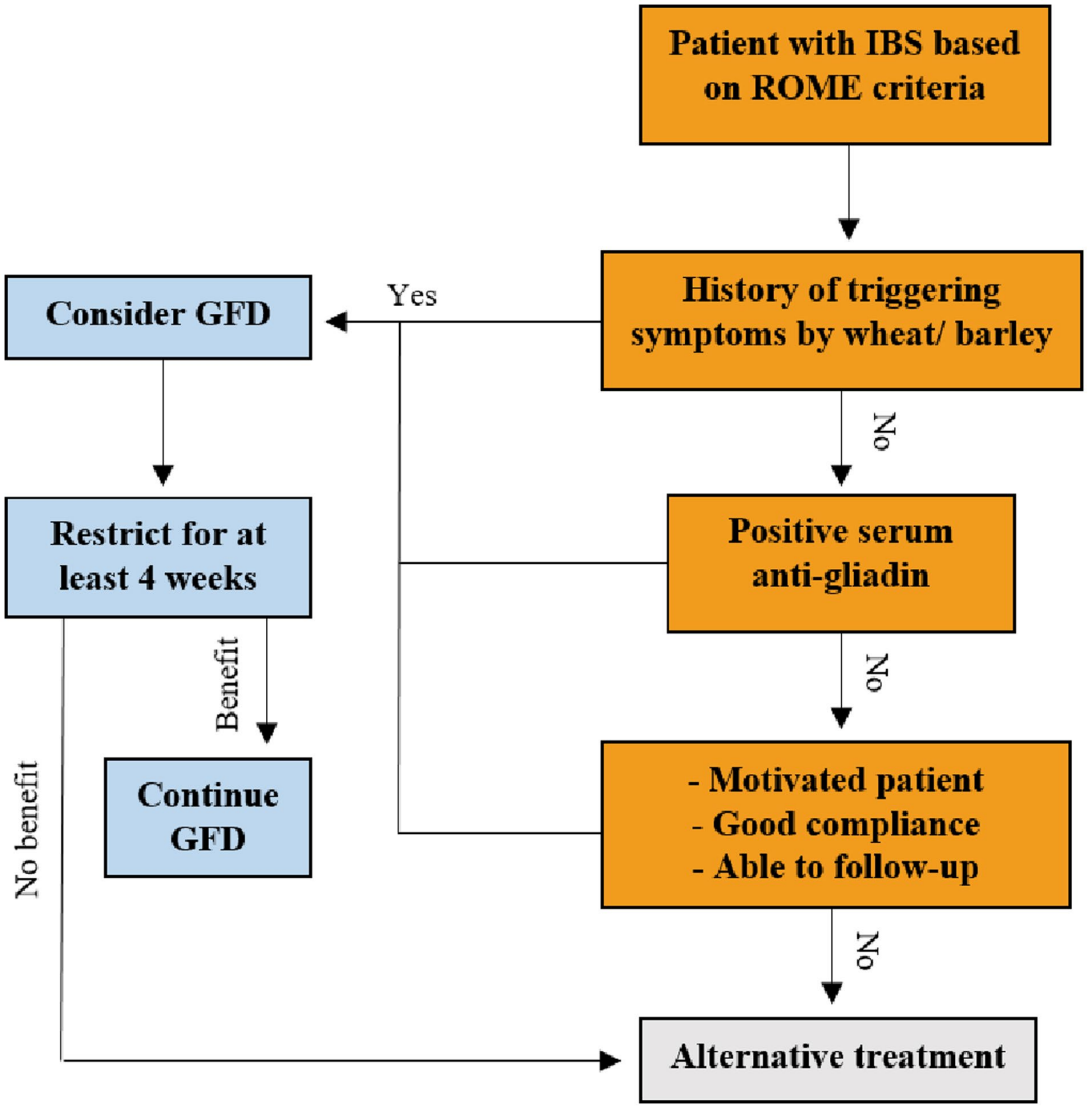
Suggested algorithm for consideration of GFD for IBS.

On the other hand, while pooled data from seven studies showed no effect for GFD on overall symptoms, subgroup analysis revealed a significant effect for more than 4 weeks of intervention and a significant between subgroup heterogeneity (*p* = 0.011). Thus, it seems the length of intervention has a crucial role in the effectiveness of GFD. However, the presence of only two studies in this subgroup makes it difficult to draw a firm conclusion. The data are generally controversial, but at least 4 weeks of GFD may be needed to evaluate its impact on IBS symptoms. The suggested algorithm for consideration of GFD for IBS is illustrated in Figure 4.

The present meta-analysis appears to contain several strengths and limitations. This study, to the best of our knowledge, is the first systematic review and meta-analysis evaluates the effect of GFD on the spectrum of GI symptoms of IBS and compares it with the LFD. The number of eligible studies has also extended remarkably since the topic was last evaluated. Finally, based on the GRADE guidelines, we rigorously evaluated the certainty of evidence across the studies. However, some limitations of this study should be taken into consideration. The relatively small number of trials in some effect sizes diminishes the robustness of the results. This applies more clearly in studies compared GFD with LFD, as there were just two studies in this group. The analysis revealed a high statistical heterogeneity. This may be due to the diversity of methodologies (different designs), differences in intervention type (GFD, GCD, LFD, TDA, and other types of diet) or intervention length (1 to 12 weeks), diagnostic criteria (ROME III or IV), and IBS subtype (constipation, diarrhea, or mixed). Moreover, almost half of the eligible studies were from Iran, limiting the study to reflect diverse global populations and generalizing the results. Also, as with any meta-analysis, limitations associated with potential publication bias should be regarded. Finally, due to insufficient or lack of data, this study was unable to evaluate the efficacy of GFD based on age, sex, or predominant stool type subgroups; determine the precise predictors of response to GFD; and compare the impact of GFD alone with its combination with the other diets. More studies are needed to fill this gap.

## 5. Conclusion

This is the first meta-analysis to assess the effects of GFD on IBS symptoms to this extent. The findings showed that a GFD is not robust enough to be a routine recommendation for IBS patients. Furthermore, GFD efficacy is significantly lower than that of an LFD. This diet might be beneficial for just a specific subgroup of IBS patients; further studies are needed to target this subgroup.

## Data availability statement

The original contributions presented in the study are included in the article/Supplementary material, further inquiries can be directed to the corresponding authors.

## Author contributions

EA: Conceptualization, Data curation, Formal analysis, Investigation, Methodology, Software, Validation, Writing – original draft, Writing – review & editing. DA: Conceptualization, Data curation, Writing – original draft, Writing – review & editing. AS: Conceptualization, Investigation, Methodology, Project administration, Supervision, Validation, Writing – review & editing. SK: Data curation, Visualization, Writing – original draft. AH: Conceptualization, Methodology, Supervision, Writing – review & editing. HK-V: Data curation, Formal Analysis, Methodology, Software, Validation, Writing – review & editing. MA: Supervision, Writing – review & editing.
