# Supplementary material for: Gluten restriction in irritable bowel syndrome, yes or no?: a GRADE-assessed systematic review and meta-analysis
Source: Front Nutr. 2023 Nov 1;10:1273629. doi: 10.3389/fnut.2023.1273629 (PMC10646307; doi:10.3389/fnut.2023.1273629)
Supplement: Supplementary file 1 [file Data_Sheet_1.DOCX]

**Table S1.** Search terms used across the main databases

| **Database** | **Search strategy** |
| --- | --- |
| **Pubmed** | ((IBS[Title/Abstract]) OR (irritable bowel syndrome[Title/Abstract]) OR (irritable colon[Title/Abstract]) OR (spastic colon[Title/Abstract])) AND ((Gluten[Title/Abstract]) OR (gluten-free[Title/Abstract]) OR (GFD[Title/Abstract])) |
| **Scopus** | ( ( TITLE-ABS-KEY ( gluten )  OR  TITLE-ABS-KEY ( gluten-free )  OR  TITLE-ABS-KEY ( gfd ) ) )  AND  ( ( TITLE-ABS KEY ( irritable  AND bowel  AND syndrome )  OR  TITLE-ABS-KEY ( ibs )  OR  TITLE-ABS-KEY ( irritable  AND colon )  OR  TITLE-ABS-KEY ( spastic  AND colon ) ) ) |

**Table S2.** Excluded studies in full-text review

| **Reason for exclusion** | **Reference** |
| --- | --- |
| Inappropriate design (n=3) | (1–3) |
| Cross-sectional study (n=2) | (4,5) |
| Focused on non-celiac gluten sensitivity (n=2) | (6,7) |
| Without clinical data (n=1) | (8) |
| Without control arm (n=1) | (9) |

**Table S3.** Influence analysis of GFD effects on overall symptoms

| **Study omitted** | **Estimate** | **[95% Conf.** | **Interval]** |
| --- | --- | --- | --- |
| Algera (2022) | -.30756477 | -1.0259459 | .41081631 |
| Nordin (2022) | -.49521697 | -1.0918037 | .10136977 |
| Paduano (2019) | -.41999742 | -1.1298966 | .28990185 |
| Rej (2022) | -.4033432 | -1.1320851 | .32539865 |
| Mohseni (2022) | -.14631188 | -.76085103 | .46822724 |
| Saadati (2022) | -.23364538 | -.90991527 | .44262448 |
| Zanwar (2016) | -.14757764 | -.75259554 | .45744023 |
| **Combined** | **-.30765975** | **-.92492953** | **.30961004** |

**Table S4.** Influence analysis of GFD effects on abdominal pain

| **Study omitted** | **Estimate** | **[95% Conf.** | **Interval]** |
| --- | --- | --- | --- |
| Algera (2022) | -.68187839 | -1.3605109 | -.00324589 |
| Hajoani (2019) | -.68187839 | -1.3605109 | -.00324589 |
| Mohseni (2022) | -.71257406 | -1.4794706 | .05432246 |
| Paduano (2019) | -.80809331 | -1.6482387 | .03205198 |
| Rej (2022) | -.70510787 | -1.478941 | .06872527 |
| Saadati (2022) | -.79282105 | -1.5791227 | -.00651944 |
| Shahbazkhani (2015) | -.75864637 | -1.5586144 | .04132157 |
| Zanwar (2016) | -.6329549 | -1.3647835 | .09887368 |
| **Combined** | **-.68187841** | **-1.3605109** | **-.00324589** |

**Table S5.** Influence analysis of GFD effects on bloating

| **Study omitted** | **Estimate** | **[95% Conf.** | **Interval]** |
| --- | --- | --- | --- |
| Algera (2022) | -.41117537 | -1.213824 | .39147326 |
| Rej (2022) | -.54486459 | -1.3045011 | .21477179 |
| Mohseni (2022) | -.0256925 | -.45769221 | .40630722 |
| Shahbazkhani (2015) | -.51459807 | -1.3190802 | .28988403 |
| Paduano (2019) | -.4538244 | -1.28487 | .3772212 |
| Saadati (2022) | -.23215441 | -.93871802 | .47440919 |
| **Combined** | **-.36658414** | **-1.0299025** | **.29673419** |

**Table S6.** Risk of bias assessment based on Cochrane tool

| **Reference** | **Random sequence generation** | **Allocation concealment** | **Selective reporting** | **Other sources of bias** | **Blinding(participants and personnel)** | **blinding(outcome assessment)** | **Incomplete outcome data** | **General risk of bias** |
| --- | --- | --- | --- | --- | --- | --- | --- | --- |
| Shahbazkhani (2015)(10) | L | L | L | H | L | L | H | **H** |
| Algera (2022)(11) | L | L | L | L | L | L | L | **L** |
| Hajiani (2019)(12) | H | L | H | L | L | L | H | **H** |
| Nordin (2022)(13) | L | L | L | L | L | L | L | **L** |
| Paduano (2019)(14) | H | L | L | L | L | U | L | **L** |
| Rej (2022)(15) | L | L | L | L | L | L | L | **L** |
| Saadati (2022)(16) | U | L | L | L | L | L | L | **L** |
| Zanwar (2016) (17) | L | L | H | L | L | L | H | **H** |
| Mohseni (2022)(18) | U | L | L | L | L | L | L | **L** |

**Figure S1.** Funnel plots for publication bias

Overall symptoms


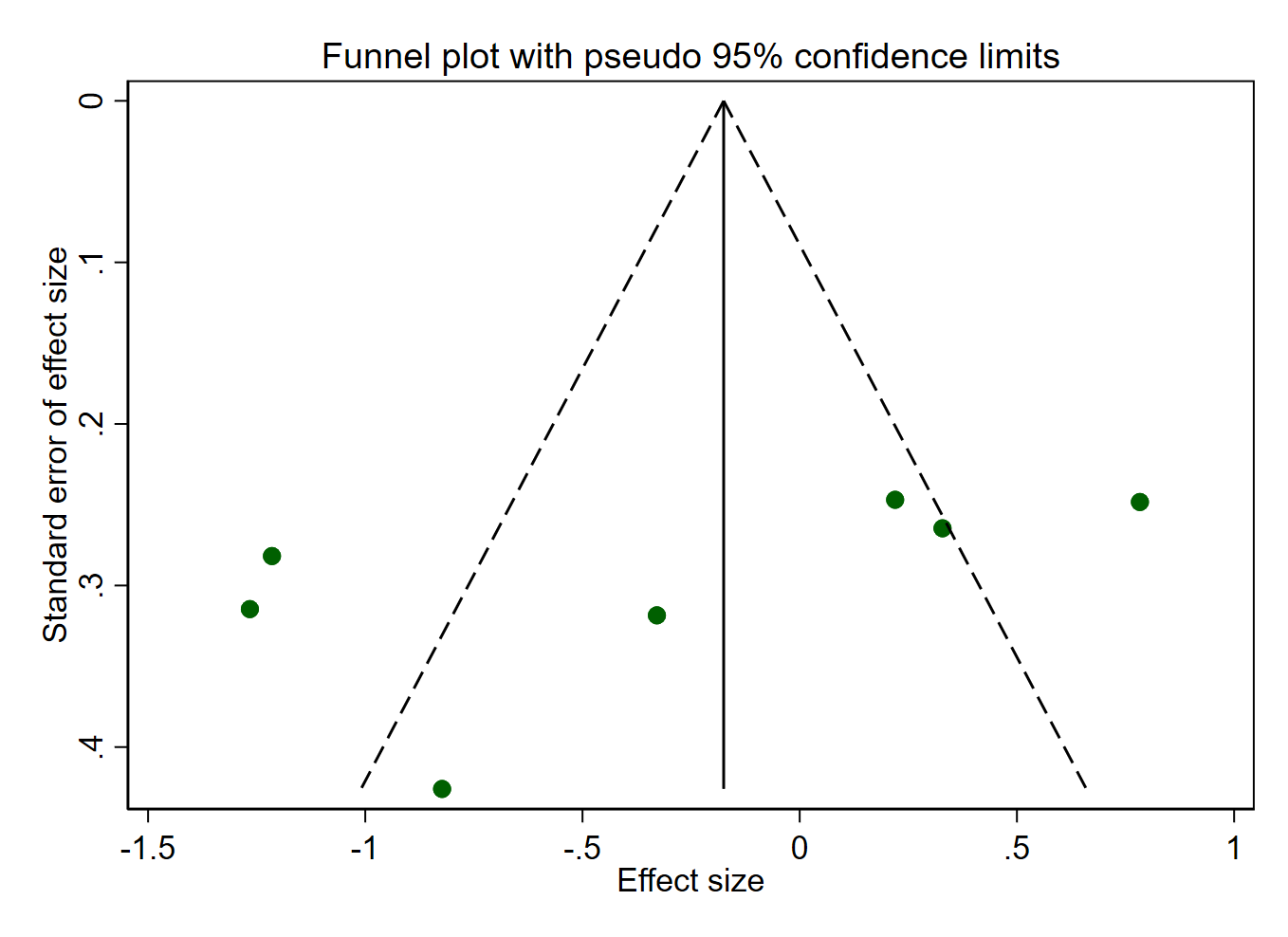


Abdominal pain


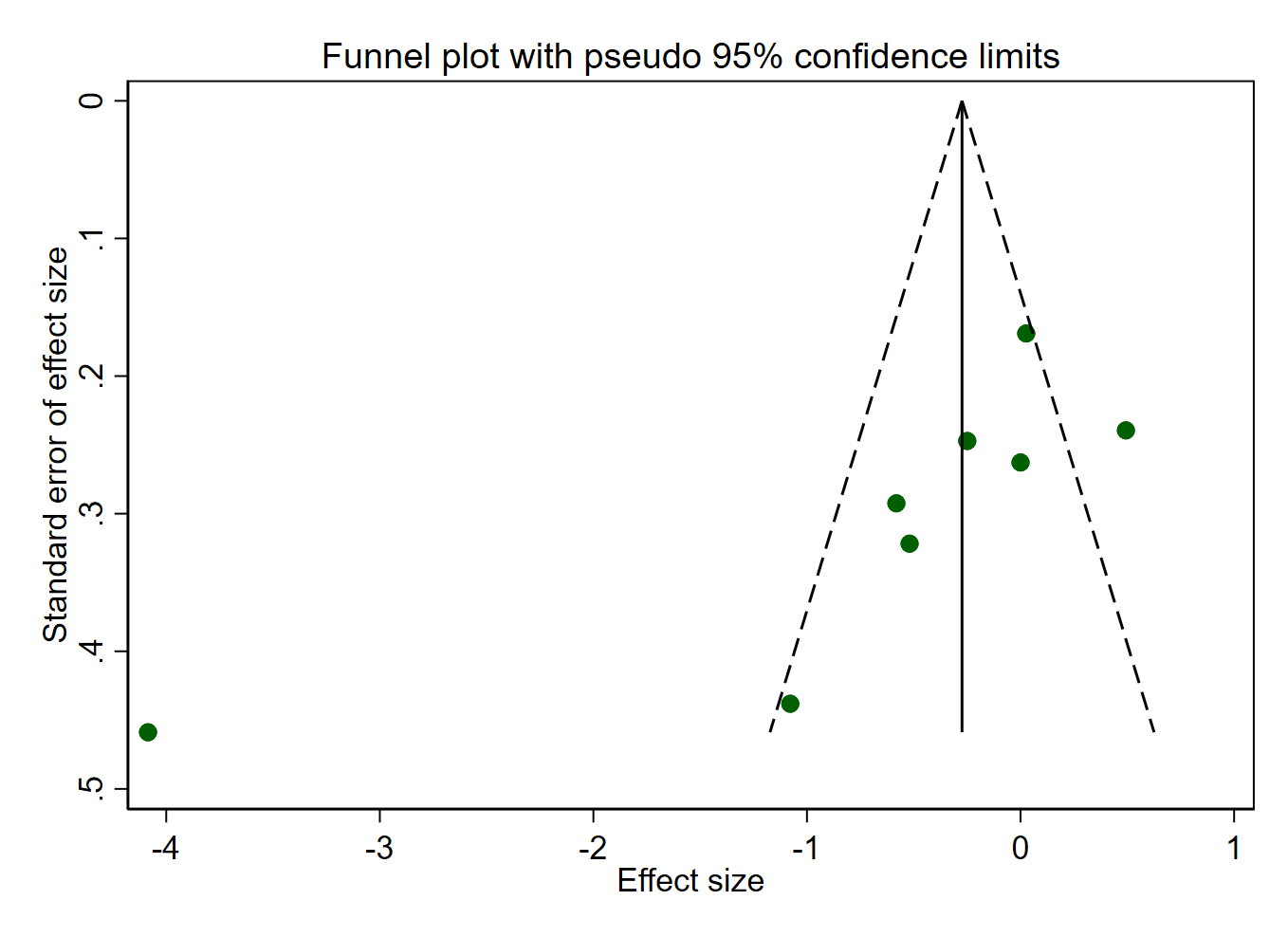


Bloating


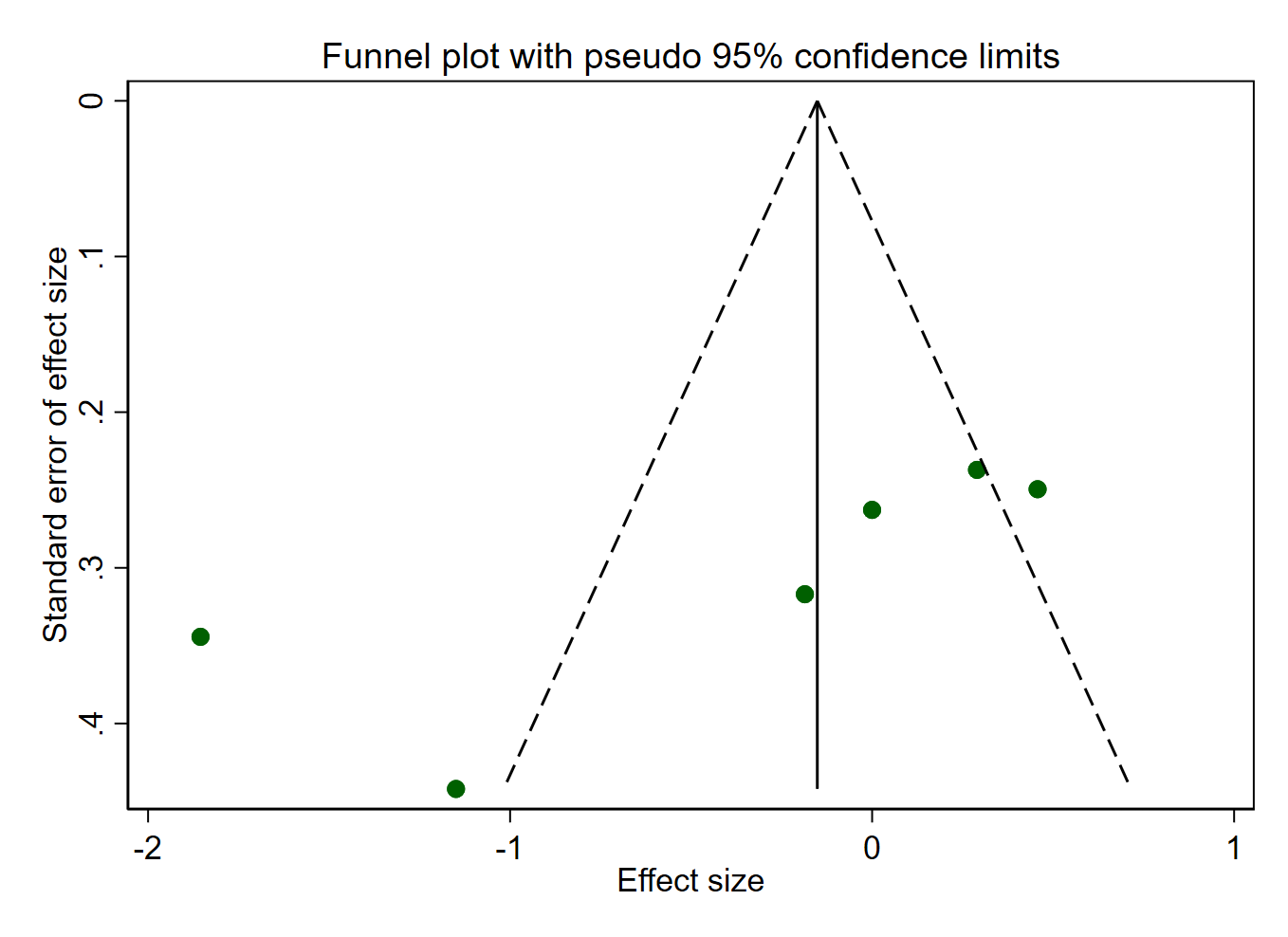


**Visual abstract**

**
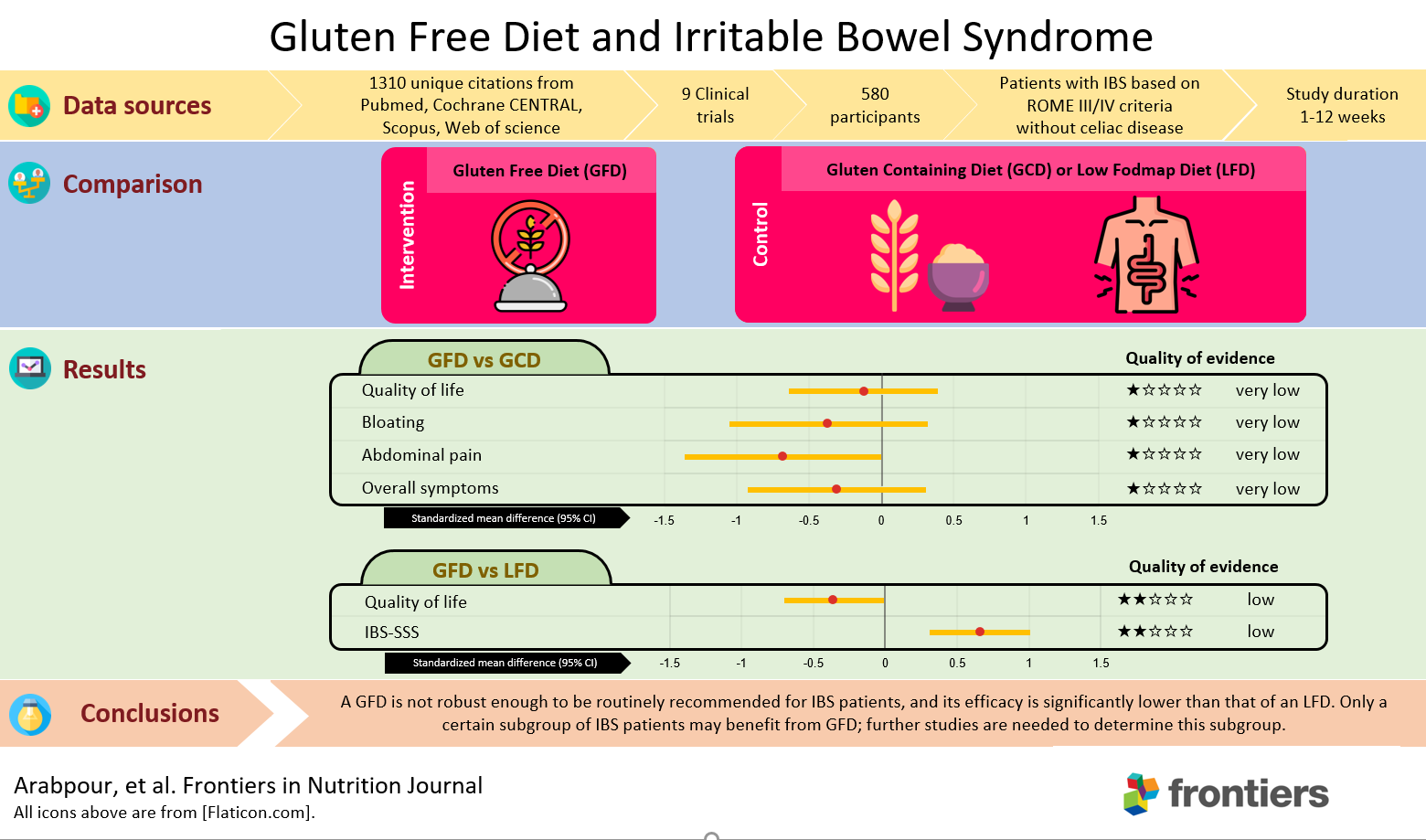
**

1. Barone M, Gemello E, Viggiani MT, Cristofori F, Renna C, Iannone A, et al. Evaluation of non-celiac gluten sensitivity in patients with previous diagnosis of irritable bowel syndrome: A randomized double-blind placebo-controlled crossover trial. Nutrients. 2020 Mar 6;12(3):705.

2. Biesiekierski JR, Newnham ED, Irving PM, Barrett JS, Haines M, Doecke JD, et al. Gluten causes gastrointestinal symptoms in subjects without celiac disease: a double-blind randomized placebo-controlled trial. Am J Gastroenterol. 2011 Mar;106(3):508–14; quiz 515.

3. Biesiekierski JR, Peters SL, Newnham ED, Rosella O, Muir JG, Gibson PR. No effects of gluten in patients with self-reported non-celiac gluten sensitivity after dietary reduction of fermentable, poorly absorbed, short-chain carbohydrates. Gastroenterology. 2013 Aug;145(2):320-8.e1-3.

4. Algera JP, Störsrud S, Lindström A, Simrén M, Törnblom H. Gluten and fructan intake and their associations with gastrointestinal symptoms in irritable bowel syndrome: A food diary study. Clin Nutr. 2021 Oct;40(10):5365–72.

5. Barmeyer C, Schumann M, Meyer T, Zielinski C, Zuberbier T, Siegmund B, et al. Long-term response to gluten-free diet as evidence for non-celiac wheat sensitivity in one third of patients with diarrhea-dominant and mixed-type irritable bowel syndrome. Int J Colorectal Dis. 2017 Jan;32(1):29–39.

6. Skodje GI, Minelle IH, Rolfsen KL, Iacovou M, Lundin KEA, Veierød MB, et al. Dietary and symptom assessment in adults with self-reported non-coeliac gluten sensitivity. Clin Nutr ESPEN. 2019 Jun;31:88–94.

7. Skodje GI, Sarna VK, Minelle IH, Rolfsen KL, Muir JG, Gibson PR, et al. Fructan, rather than gluten, induces symptoms in patients with self-reported non-celiac gluten sensitivity. Gastroenterology. 2018 Feb;154(3):529-539.e2.

8. Ajamian M, Rosella G, Newnham ED, Biesiekierski JR, Muir JG, Gibson PR. Effect of gluten ingestion and FODMAP restriction on intestinal epithelial integrity in patients with irritable bowel syndrome and self-reported non-coeliac gluten sensitivity. Mol Nutr Food Res. 2021 Mar;65(5):e1901275.

9. Naseri K, Dabiri H, Rostami-Nejad M, Yadegar A, Houri H, Olfatifar M, et al. Influence of low FODMAP-gluten free diet on gut microbiota alterations and symptom severity in Iranian patients with irritable bowel syndrome. BMC Gastroenterol. 2021 Jul 14;21(1):292.

10. Shahbazkhani B, Sadeghi A, Malekzadeh R, Khatavi F, Etemadi M, Kalantri E, et al. Non-celiac gluten sensitivity has narrowed the spectrum of irritable bowel syndrome: A double-blind randomized placebo-controlled trial. Nutrients. 2015 Jun 5;7(6):4542–54.

11. Algera JP, Magnusson MK, Öhman L, Störsrud S, Simrén M, Törnblom H. Randomised controlled trial: effects of gluten-free diet on symptoms and the gut microenvironment in irritable bowel syndrome. Aliment Pharmacol Ther. 2022 Nov;56(9):1318–27.

12. Hajiani E, Masjedizadeh A, Shayesteh AA, Babazadeh S, Seyedian SS. Comparison between gluten-free regime and regime with gluten in symptoms of patients with irritable bowel syndrome (IBS). J Family Med Prim Care. 2019 May;8(5):1691–5.

13. Nordin E, Brunius C, Landberg R, Hellström PM. Fermentable oligo-, di-, monosaccharides, and polyols (FODMAPs), but not gluten, elicit modest symptoms of irritable bowel syndrome: a double-blind, placebo-controlled, randomized three-way crossover trial. Am J Clin Nutr. 2022 Feb 9;115(2):344–52.

14. Paduano D, Cingolani A, Tanda E, Usai P. Effect of three diets (low-FODMAP, gluten-free and balanced) on Irritable Bowel Syndrome symptoms and health-related quality of life. Nutrients. 2019 Jul 11;11(7):1566.

15. Rej A, Sanders DS, Shaw CC, Buckle R, Trott N, Agrawal A, et al. Efficacy and acceptability of dietary therapies in non-constipated irritable bowel syndrome: A randomized trial of traditional dietary advice, the low FODMAP diet, and the gluten-free diet. Clin Gastroenterol Hepatol. 2022 Dec;20(12):2876-2887.e15.

16. Saadati S, Sadeghi A, Mohaghegh-Shalmani H, Rostami-Nejad M, Elli L, Asadzadeh-Aghdaei H, et al. Effects of a gluten challenge in patients with irritable bowel syndrome: a randomized single-blind controlled clinical trial. Sci Rep. 2022 Mar 23;12(1):4960.

17. Zanwar VG, Pawar SV, Gambhire PA, Jain SS, Surude RG, Shah VB, et al. Symptomatic improvement with gluten restriction in irritable bowel syndrome: a prospective, randomized, double blinded placebo controlled trial. Intest Res. 2016 Oct;14(4):343–50.

18. Mohseni F, Agah S, Ebrahimi-Daryani N, Taher M, Nattagh-Eshtivani E, Karimi S, et al. The effect of low FODMAP diet with and without gluten on irritable bowel syndrome: A double blind, placebo controlled randomized clinical trial. Clin Nutr ESPEN. 2022 Feb;47:45–50.
